# Melanopsin-Mediated Acute Light Responses Measured in Winter and in Summer: Seasonal Variations in Adults with and without Cataracts

**DOI:** 10.3389/fneur.2017.00464

**Published:** 2017-09-11

**Authors:** Mirjam Münch, Myriam Ladaique, Ségolène Roemer, Kattayoon Hashemi, Aki Kawasaki

**Affiliations:** ^1^Group Sleep Research & Clinical Chronobiology, Institute of Physiology, Charité University Médicine Berlin, Berlin, Germany; ^2^Hôpital Ophtalmique Jules Gonin, University of Lausanne, Lausanne, Switzerland

**Keywords:** pupil, cataract, melanopsin, melatonin, intrinsically photosensitive retinal ganglion cell, post-illumination pupil response, daylight

## Abstract

Seasonal adaptation is a ubiquitous behavior seen in many species on both global hemispheres and is conveyed by changing photoperiods. In humans this seasonal adaptation is less apparent, in part because changes in daylength are masked by the use of electrical lighting at night. On the other hand, cataracts which reduce light transmission, may compound seasonal changes related to the reduced daylength of winter. To better understand the effects of different photoperiod lengths in healthy adults without and with cataracts, we tested their melanopsin-mediated light responses in summer vs. winter. Fifty-two participants (mean age 67.4 years; 30 with bilateral cataracts and 22 age-matched controls with clear lenses; pseudophakes) were tested twice, once in summer and once in winter. At each test session we assessed the electroretinogram and pupil responses during daytime and we determined melatonin suppression, subjective sleepiness and mood in response to light exposure in the evening. Circadian rest-activity cycles and sleep from activity recordings were also analyzed for both seasons. Both groups had similar visual function. There were no seasonal differences in the electroretinogram. For the pupil responses to bright blue light, the post-illumination pupil response (PIPR) was greater in winter than summer in pseudophakes, but not in cataract participants, whereas melatonin suppression to acute light exposure showed no differences between both groups and seasons. Overall, intra-daily variability of rest-activity was worse in winter but participants felt sleepier and reported worse mood at the laboratory in evening time in the summer. Those with cataracts had poorer sleep quality with lower sleep efficiency, and higher activity during sleep in winter than summer. In this study, the PIPR showed a seasonal variation in which a larger response was found during winter. This variation was only detected in participants with a clear intraocular lens. In the cataract group, visual function was not impaired yet these participants showed a lack of seasonal changes in the pupil response to blue light and poorer sleep in winter. These findings raise the question for tailored lighting conditions for cataract patients in order to counter potentially deleterious effects of living with chronically lower light exposure.

## Introduction

The spectral composition, intensity, and duration of environmental light vary with time of day and across the seasons (1). The seasonal variation in daylight duration, or photoperiod, is a strong environmental cue for inducing various adaptive changes in the physiology and behavior of most animals to the different seasons. These adaptations include changes in food gathering, migration, hibernation, reproduction, and even immune responsiveness (2–6).

The influence of changing seasonal light and photoperiod is less notable in humans compared with animals, perhaps in part related to the artificial lighting conditions of human habitation (7–12). Nevertheless, some degree of a change in mood and behavior across the seasons (seasonality) is experienced in the general population (7). A genetic predisposition may make one subject more or less responsive to seasonal daylight changes and may underlie adaptive responses to stress (12). In some humans, however, a shortened photoperiod is associated with a maladaptive pathologic response; a condition termed seasonal affective disorder (SAD) (13, 14). SAD is characterized by depressive episodes which often occur in the fall or winter and remit in the spring or summer (13). While the pathophysiologic basis for SAD has yet to be defined, emerging evidence suggests that differences in ocular light perception may have relevance in this disorder (15–19).

The primary photoreceptor for detecting environmental light is the intrinsically photosensitive retinal ganglion cell (ipRGC) which expresses the photopigment melanopsin and is located in the inner retina (20–22). In mammals, one of the primary synaptic sites of ipRGCs is the hypothalamic suprachiasmatic nucleus (SCN) the master pacemaker which synchronizes the internal circadian clocks to the temporal changes in environmental light (23). The SCN adapts to diurnal and seasonal changes by modulating neurotransmitters and clock gene expression which lead to changes of its rhythmic electrical firing patterns (6). The rhythm of the SCN is then conveyed to other brain areas and peripheral organs. One of the best known indirect circadian markers of the internal clock is melatonin secretion in the pineal gland.

Suppression of nocturnal melatonin secretion by acute light exposure (24) is a reliable marker of light input to the hypothalamus. Melatonin suppression is mediated by ipRGCs whose activity is driven primarily by melanopsin activation with contribution from rods and cones. Melatonin suppression can be quantified by a dose–response relationship based on exposure duration, light intensity, and spectral composition of light (25–29). IpRGCs also have direct synaptic input to the pre-tectum of the dorsal midbrain which is an important integrating site for inputs to the pupil light reflex (30–32). Based on cellular responses to light in animal studies, several human-based investigations have demonstrated that in pupil responses to light, intrinsic melanopsin activity is best observed in the post-light dynamics (33–35). Melanopsin activity progressively delays pupil re-dilation of the dark-adapted pupil and leads to a more sustained state of pupillary constriction following termination of the light stimulus, termed the “post-illumination pupil response” (PIPR) (34, 36, 37).

In wild-type mice, it has been demonstrated that abnormal light exposure patterns will induce depression-like behavior which leads to learning deficits but does not disrupt sleep distribution across 24 h or sleep architecture. Melanopsin knockout mice however, did not show these aberrations, indicating that light effects on mood are directly associated with light/dark exposure patterns conveyed by ipRGC (38). In humans, certain melanopsin gene polymorphisms may be associated with a greater sensitivity to light, thereby conferring to earlier (or later) sleep onset. This suggests that functional differences in ipRGC activity contribute to inter-individual differences in sleep and alertness during shortened photoperiods, such as winter months (39, 40). In recent studies, the PIPR was noted to be reduced in patients with SAD compared with healthy controls, prompting conjecture that reduced melanopsin sensitivity that is particularly notable during winter might be involved in the pathogenesis of the disease (41, 42). At the molecular level, a missense allele in the melanopsin gene found in SAD patients has been suggested to contribute to this change in melanopsin sensitivity (39). One hypothesis for the pathophysiologic basis of SAD is reduced retinal light sensitivity from a failure to increase light sensitivity during winter (43–45). First evidence has emerged in a recent study by Roecklein et al., who indeed found in SAD patients compared with controls a reduced PIPR in winter but not in summer (46). This would imply that the “normal” retinal function includes seasonal variation as part of a variety of adaptive changes related to changing photoperiods. Assessment via the pupil has demonstrated variation in ipRGC activity, i.e., variation in responses to bright blue-light stimuli that is both diurnal and seasonal (42, 47–49).

This study aimed to assess for seasonal variation in the responsiveness of the melanopsin system as measured via pupillary, hormonal, and behavioral responses to acute light exposure. ipRGC stimulation by retinal light influx is the common point for melanopsin-dependent pathways which contributes to acute light responses. Thus, we endeavored to assess the PIPR and melatonin suppression as well as subjective sleepiness in healthy adults during winter and summer. In addition, we wished to understand if chronically reduced light exposure might influence these responses differently, particularly in winter when ambient light levels are lower. Conditions which lead to chronically (year-long) reduced light exposure such as confined lifestyles or certain ocular disorders can influence other non-visual functions, e.g., circadian rhythms (50), melatonin suppression (51), and sleep (52–54) as well as cognitive performance such as reaction time (55, 56). Age-related yellowing and opacification of the intraocular lens (cataracts) impede light transmission to the retina (57, 58), particularly that of short wavelength, and could serve as a model for chronically reduced light exposure in the context of otherwise healthy eyes and modern lifestyle. A better understanding of the influence of chronically reduced light exposure on human well-being might suggest ways to tailor lighting strategies for patients with ocular disorders and for persons living under low light (natural and artificial) conditions.

## Materials and Methods

### Participants

Participants were recruited following chart reviews from the general clinic at the Hôpital Ophtalmique Jules Gonin in Lausanne, Switzerland (geographic latitude: 46° 31’ N). Fifty-two healthy adults with either bilateral cataracts or bilateral pseudophakia (i.e., after lens replacement) were invited to participate in the study. Pseudophakes with blue-blocking implants were excluded. In addition, based on a screening medical questionnaire, subjects were excluded for any of the following conditions: ocular disease other than refractive error, diabetes, cancer, sleep apnea, pregnant state during the study, known psychiatric disorder, excessive consumption of alcohol or tobacco, use of sedatives, melatonin, opioids, night shift work, and travel across more than one time zone in the month before study testing. Six subjects were taking beta blockers on a regular basis (four cataracts and two pseudophakes). The study was conducted according to the tenets of the Declaration of Helsinki and received authorization from the local ethical board committee for human research for the canton of Vaud of Switzerland. All study participants provided oral and written informed consent.

### Screening Examination

All participants completed five questionnaires (see below) and underwent a baseline ophthalmologic examination which included best-corrected visual acuity, color vision testing with the Ishihara book, slit lamp examination, and funduscopy. Visual field of the central 30° was assessed using threshold automated perimetry (Octopus 101, Interzeag, Bern-Köniz, Switzerland). The macula and peripapillary retinal nerve fiber layer were examined by optical coherence tomography (Stratus 3000, Carl Zeiss, Meditec, Inc., Dublin, CA, USA). The pseudophakic subjects had undergone bilateral cataract surgery with intraocular clear (non-blue-blocking) lens replacement within 5 months of inclusion in the study. Participants with cataracts had slit lamp photography of the lens, through a dilated pupil, taken by a certified ocular photographer. The nuclear color and opalescence of the cataract was graded by a cataract specialist on a scale from 1 to 6 where a grade > 2 is considered clinically relevant (59).

### Screening Questionnaires

In order to assess the chronotype of all subjects, the Horne–Ostberg (HO) Questionnaire as well as the Munich Chronotype Questionnaire (MCTQ) was administered. The latter assesses the habitual midpoint of sleep, corrected for sleep duration on free days (MSF_SC_). The Pittsburgh Sleep Quality Index (PSQI), an indicator for sleep disturbances, was also employed. A global PSQI score ≤5 usually indicates normal sleep. The Beck Depression Inventory (BDI) is a 21-question multiple-choice self-report inventory used for screening symptoms related to depression. The Seasonal Pattern Assessment Questionnaire (SPAQ) was used to estimate seasonal changes in sleep and eating habits as well as in mood and behavior. The global seasonality score (GSS) of the SPAQ is a composite score which indicates the degree to which sleep, weight, mood, social activity, appetite, and energy are affected by season; a higher score indicates greater changes in these attributes between seasons. No participant was excluded based on a questionnaire score.

### Study Design

Participants were scheduled for two testing sessions during two photoperiods within 12 consecutive months. The first period included the 6 weeks before and after the winter solstice, while the second period comprised the 6 weeks before and after the summer solstice. Each seasonal testing session consisted of a day protocol and a night protocol on separate days.

The day testing was performed between 8 a.m. and 5 p.m. First, participants underwent monocular and binocular pupillometry. Thereafter, pupils were pharmacologically dilated using phenylephrine 2.5% and tropicamide 0.5% in each eye, and an electroretinogram (ERG) was performed as described below.

In the 7 days prior to the evening testing, participants were at home and exposed to their usual environmental lighting conditions. They were instructed to maintain a regular sleep/wake cycle with approximately 8 h in bed per night at the same self-selected bed- and wake times, and to refrain from napping during daytime. Rest-activity cycles were monitored by activity watches and sleep logs.

For the evening testing session, participants arrived at the laboratory 5 h before their habitual bedtime. They were asked to refrain from ingesting food or drinks containing caffeine or alcohol during the day of the evening testing. At the laboratory, illumination in a vertical direction of the eye was steadily maintained at <6 lx, except during the period of experimental light exposure (see below). During the entire evening testing, participants remained seated and were permitted to read, listen to music or engage in conversation. The research assistant ensured that participants did not fall asleep or utilize external light sources. Light snacks and beverages were offered at regular intervals. Salivary samples for determination of melatonin concentration as well as assessments of subjective sleepiness by questionnaire were executed hourly, including before and after the experimental light exposure (see below).

### Study Tests and Outcome Measures

#### Electroretinogram

In order to assess outer retinal function, full-field electrodiagnostic testing was performed on dilated pupils with a portable device (RETIcom, Roland Consult, Brandenburg, Germany) with a mini-Ganzfeld monocular stimulator and microfiber silver thread recording electrodes placed bilaterally along the internal and external canthi. The ERG was recorded from both eyes, starting with the right eye. The photopic sequence was performed following 5 min of room light adaptation (~90 lx in a vertical direction of the eyes). The first sequence consisted of 20 white light flashes at an intensity of 0 dB (3 cd/m^2^) and the second sequence used a 30 Hz flicker at 0 dB. Following this, the participants were dark adapted (0 lx) for 30 min and two scotopic sequences are performed: eight white light flashes at −25 dB, followed by eight white light flashes at 0 dB.

The recordings were averaged and plotted as a function of time. The b-wave amplitude (microvolts) and implicit time (milliseconds) to −25 dB of polychromatic white light under dark adaptation was selected as a functional measure of rod activity. The b-wave amplitude and implicit time as well as flicker time to 0 dB of polychromatic white light under light adaptation were used to measure cone activity.

#### Pupillometry

Computerized pupillography was performed during daytime hours under conditions of dark and light adaptation. Details of the instrumentation have been described previously (60). In brief, a ColorDome Ganzfeld ERG apparatus (Diagnosys, Lowell, MA, USA) was used to present a 1 s light stimulus having red color (640 ± 10 nm) or blue color (467 ± 17 nm) at pre-selected intensities to one or both eyes. Light intensities in this study ranged from −4.0 to 2.3 log cd/m^2^ (0 log = 1 cd/m^2^). A dual channel binocular pupillometer mounted on an eye frame (Arrington Research, Scottsdale, AZ, USA) continuously recorded the pupil diameter at 60 Hz for the duration of each pupil test, resulting in >2,000 data points per tracing, depending on the light sequence. Four different light sequences were used in this study in order to preferentially stimulate the rods vs. cones vs. melanopsin cells.

The light sequences used were named for the prevailing photoreceptor targeted. The sequence order was as follows: rod-weighted sequence, cone-weighted sequence, melanopsin sequence with monocular stimulation, and melanopsin sequence with binocular stimulation. The rod-weighted sequence was preceded by 10 min of dark adaptation (0 lx). Six dim blue-light stimuli were presented at half-log unit steps of increasing intensity (−4, −3.5, −3, −2.5, −2, −1.5 log cd/m^2^, respectively). The inter-stimulus interval ranged from 3 to 7 s, having been previously determined in order to permit the pupil to return to baseline size. The cone-weighted sequence was preceded by 10 min of polychromatic white light adaptation (room light ~90 lx at the vertical eye level). The pupil was recorded for 5 s in darkness before the first red-light stimulus (0 log cd/m^2^) was presented. Following this, another four stimuli of increasing intensity (1, 1.5, 2, 2.5 log cd/m^2^, respectively) were presented. The inter-stimulus interval was 3, 7, 10, and 20 s, respectively. The melanopsin-weighted sequence with monocular stimulation was preceded by 10 min of room light adaptation (~90 lx). Three bright blue-light stimuli at 1, 1.5, and 2 log cd/m^2^ were presented. The inter-stimulus intervals were sufficiently long, 25 and 35 s, respectively, in order to permit the pupil to recover to baseline size. The melanopsin-weighted sequence with binocular stimulation followed the monocular sequence. It consisted of a single red flash (2.3 log cd/m^2^) followed 30 s later by a single bright blue-light stimulus (2.3 log cd/m^2^). For the two melanopsin-weighted protocols, the last light stimulus was followed by 35 and 60 s of pupil recording in darkness for the monocular and binocular recordings, respectively, in order to record the post-stimulus pupillary dynamics.

The pupil data were exported to a PC for analysis. The analysis was done by means of a customized script [developed by Michael Philipp Notter, Lausanne, Switzerland (61)] that was executed with the programming software GNU Octave version 3.0.1 (62). The script removed blink artifacts from the raw pupil tracings with a customized semi-automated filter function. The baseline pupil size was the average size during the 0.25 s prior to each light stimulus; thereafter, pupil size at any given time was converted to relative pupil size. The maximum contraction amplitude in response to a light stimulus was identified as the smallest pupil size within 3 s after light stimulus onset and having a latency of least 500 ms. The maximal contraction amplitude was expressed in percentage as the difference from the baseline pupil size by applying the following equation: [Maximum contraction amplitude = (1 − smallest relative pupil size) × 100%].

A criterion level of 4% in contraction amplitude 1/60 s was applied to distinguish evoked pupil responses from random noise. For the rod- and cone-weighted sequences, the maximal contraction amplitude in response to each light stimulus was the primary outcome parameter.

The persistence of pupillary constriction after termination of a bright blue-light stimulus is the characteristic pupillographic feature of melanopsin activation in humans and described as the PIPR (36, 63). In keeping with other studies which have evaluated the PIPR, we used a previously defined metric, namely the contraction amplitude at 6 s after termination of the light stimulus (36, 63). Post-illumination contraction amplitude at 6 s after light offset is the percentage difference from baseline pupil size at this time and was calculated by applying the equation: [Post-illumination contraction amplitude = (1 − relative pupil size at 6 s after light offset) × 100%] (36, 63). For this study, we use the abbreviation PIPR to indicate the pupil contraction amplitude at 6 s after termination of a bright blue-light stimulus.

#### Melatonin Sampling and Nocturnal Light Exposure

Salivary samples for melatonin assays were obtained by using salivettes with cotton swabs (Salivettes^®^; Sarstedt AG; Eppendorf, Germany). Five melatonin samples were obtained per participant: three samples before, one during and one after light exposure (see below). The samples were refrigerated at 4°C overnight and then frozen at −20°C. After study completion, the frozen samples were sent to an external laboratory for radio-immuno-assays [RIA; Stockgrand Ltd.; University of Surrey, Guildford, UK (64)]. The inter-assay coefficients of variance, i.e., the quality measures for the RIA assay were 12.4% (low concentrations) and 8.5% (high concentrations). The intra-assay coefficients of variance were 6.9% for low, and 2.4% for high concentrations with a detection limit of 0.6 pg/ml.

Once every hour during the 5 h before habitual bedtime, a salivary sample was obtained. One hour before habitual bedtime, the participant was exposed to polychromatic white light for 30 min. The light source was a commercially available and dimmable light box (Energy Up; HF3419/01; Philips Respironics, Switzerland), mounted on a tabletop and placed at 1 m distance of the participant’s eyes. The target illuminance was 400 lx in a vertical direction, verified by a luxmeter (Amprobe LM-100, Amprobe GmBH, Glottertal, Germany) and measured at the level of the participant’s forehead. During the 30 min of light exposure, the participant was asked to keep the eyes open (except for blinking) and look toward the middle of the light box.

#### Subjective Sleepiness, Relaxation, Physical Well-being, and Mood

Subjective sleepiness, relaxation, physical well-being, and mood were assessed immediately before each salivary sample during the evening testing. A paper-based visual analog scale was used and the participants were instructed to indicate their current state of subjective sleepiness, relaxation, physical well-being, and mood by drawing a vertical line on a line between two extremes [e.g., alert = 0 mm, sleepy = 100 mm (65)].

#### Rest-Activity Cycles and Sleep before the Evening Testing

Rest-activity cycles were monitored continuously using wrist-worn activity watches (Actiwatch L, Respironics AG, Switzerland) for 7 days prior to the evening testing. Rest-activity recordings were downloaded to a PC and each 24-h recording was visually inspected in order to further edit the data: days with more than 3 h of missing data during daytime were excluded from the analysis. Missing data which were shorter than 3 h were edited with the mean activity of 24 h using the software Sleep Analysis (v7.2, Camntech, Cambridge, UK). All edited data per participant underwent the so-called “non-parametric circadian rhythm analysis” implemented in the software (Sleep Analysis v7), and developed by Van Someren et al. (66). The following outcome parameters were assessed: intra-daily variability (IV); inter-daily stability (IS); M10on = the onset time of the 10 h with greatest activity (M10) which normally occurs during daytime, as well as L5on = the onset time of the 5 h with least activity (L5), i.e., during sleep. The relative amplitude reflects the ratio of M10/L5 amplitudes, whereas the absolute activity amplitude has arbitrary units. In general, a greater IV and a lower IS as well as a lower relative amplitude depict greater rest-activity fragmentation across several days. The sleep variables were derived from rest-activity cycles and analyzed by the sleep analysis feature of the same software.

### Statistics

The Software Package SAS (SAS Institute Inc., Cary, NC, USA; v 9.3) was used for statistical analyses. For screening differences between participants with cataracts (hereafter called “cataracts”) and pseudophakic participants (hereafter called “pseudophakes”), two-tailed *t*-tests were applied. For all within-between subject analyses, linear mixed-model analysis with the factors “group” (cataracts vs. pseudophakes) and “season” (winter vs. summer) was used. If applicable, the repeated factor “light stimulus” or “time point” or “color of light stimulus” (red vs. blue) was applied. If the data were not normally distributed, a log transformation was performed. Only those participants who completed testing in winter and summer were included in the analysis. Due to technical reasons such as poor recordings or insufficient saliva, some of the pupil recordings and/or melatonin samples had to be excluded from the analysis. All together, this resulted in sample sizes between 41 and 52 participants (the exact sample sizes for each variable are stated in the text). The covariates “iris color” (dark vs. light), “sex” and “age” were included in all linear mixed-model analyses; baseline pupil size was used as covariate in the analysis of the pupil data. The covariates age and pupil size were employed on dichotomized data (from median splitting of adjusted sample sizes). *Post hoc* tests were performed using the Tukey−Kramer test (adjusted for multiple comparisons), or the effect slices from least-square means.

## Results

### Participants and Screening Results

Fifty-two participants (cataracts and pseudophakes) were included in the study (Table 1). The age ranged from 47 to 78 years with mean age = 67.4 ± 6.3 years (SD). There were 30 cataracts (16 females, 14 males) and 22 age-matched pseudophakes (16 females, 6 males). Mean age was 68.0 ± 5.5 and 66.7 ± 7.3 years for the cataract and pseudophakic groups, respectively.

**Table 1:** Demographics, screening questionnaires, and eye examination results.

	Cataracts	Pseudophake
Age (years)	68.0 (5.5)	66.7 (7.3)
Gender	16F/14M	16F/6M
MCTQ (MSF-Sc)	3.23 (0.77)	3.29 (0.76)
BDI	2.23 (2.79)	3.73 (4.39)
PSQI^a^	**5.63 (3.92)**	**3.64 (2.06)**
HO	62.20 (9.54)	58.41 (10.61)
SPAQ score^b^	6.13 (3.71)	4.32 (3.37)
VA (RE)	1.002 (0.06)	0.984 (0.17)
VA (LE)	0.988 (0.09)	1.005 (0.13)
OCT GCL (RE)	77.83 (6.96)	77.55 (7.61)
OCT GCL (LE)	77.90 (7.18)	76.19 (9.94)
OCT RNFL (RE)	89.77 (9.84)	92.36 (9.90)
OCT RNFL (LE)	87.73 (9.28)	91.86 (12.82)
Mean VA (both eyes)	0.995 (0.07)	0.994 (0.12)
Mean OCT GCL (both eyes)	77.87 (6.99)	75.30 (11.51)
Mean OCT RNFL (both eyes)	88.75 (9.14)	91.71 (9.95)
Nuclear yellowing and opalescence (RE)	2.98 (0.61)	–
Nuclear yellowing and opalescence (LE)	3.02 (0.55)	–
Octopus mean deviation RE (dB)	0.103 (2.34)	0.295 (1.92)
Octopus mean deviation LE (dB)	−0.597 (3.40)	0.277 (1.17)

*Nuclear yellowing and opalescence assessment was only performed for cataract patients (for details see Materials and Methods)*.

*MCTQ (MSF-Sc), Munich Chronotype Questionnaire (MSF-Sc, midsleep on free days, sleep duration corrected; in decimals); BDI, Becks Depression Inventory; PSQI, Pittsburgh Sleep Quality Index; HO, Horne Ostberg; SPAQ, Seasonal Pattern Assessment Questionnaire; RE, right eye; LE, left eye; VA, visual acuity; OCT, optical coherence tomography; GCL, ganglion cell layer (micrometers); RNFL, thickness or peripapillary retinal nerve fiber layer (micrometers)*.

*^a^p < 0.05 in bold*.

*^b^Trend, p = 0.07 between cataracts and pseudophakes participants; mean ± SD; n = 52*.

The Horne Ostberg (HO) and BDI questionnaires revealed no significant differences between cataracts and pseudophakes (*n* = 52; *p* > 0.16). The PSQI scores ranged from 1 to 15 for cataracts and from 1 to 8 for pseudophakes. Thirteen cataracts and four pseudophakes had a score ≥5 and mean PSQI score for cataracts was higher than that for pseudophakes, indicating a poorer sleep quality in cataracts (*p* = 0.02; for the mean values and SDs of all questionnaires see Table 1). It was noted that two cataracts and three pseudophakes were extreme morning types, i.e., they had HO scores >70. The MCTQ (midsleep on free days, sleep duration corrected) was 3.2 ± 0.8 (*p* = 0.45 between both groups). From the SPAQ, there was a trend for higher subjective seasonality in cataracts than pseudophakes (*p* = 0.07; the averaged score for both groups combined was 3.7 ± 5.4; for both groups separately see Table 1). Five cataracts and one pseudophake had a GSS > 10; all reported impaired sleep in winter, although mood was not differently affected in winter than in summer. One patient with cataracts reported moderate problems with the seasonal changes.

The ophthalmologic examination revealed no significant differences between cataracts and pseudophakes (*p* ≥ 0.2; Table 1). Visual acuity was on average 1.0 for both groups. The cataract grade was mild-to-moderate with a mean score of 3 on the Lens Opacities Classification System III scale and ranged for the right and left eye for nuclear yellowing and opalescence: 2–5, for cortical opacity: right eye 1–3, and left eye: 1–4 and for posterior subcapsular opacity: 1–2 (see Table 1 for mean values of nuclear yellowing and opalescence; the difference between eyes was not statistically significant; *p* = 0.57).

### Electroretinogram (ERG) Results

A difference in the dark-adapted rod response (scotopic −25 dB b-wave) between cataracts and pseudophakes was noted. Cataracts showed lower amplitudes and longer implicit times than pseudophakes (main effects of group; *F*_1,48_ > 4.8; *p* < 0.04). Implicit reaction time for the scotopic 0 dB b-wave were also longer for cataracts (*F*_1,48_ = 7.6; *p* = 0.008). The photopic 0 dB a- and b-wave, and the flicker response were not significantly different between the two groups (*p* > 0.1). For cataracts there were larger photopic a- and b-wave amplitudes and faster implicit times as well as greater 30 Hz flicker amplitudes for the right than the left eye (*p* < 0.05; except for the photopic b-wave implicit time which was longer for the left than the right eye). For pseudophakes, only the photopic 0 dB b-wave implicit time was faster for the right than the left eye. None of the ERG parameters showed a seasonal variation between summer and winter (*p* > 0.07). The covariate for age was not significant for any of the measured parameters (*p* > 0.14), and the covariate sex became significant for the scotopic and photopic 0 dB a-wave amplitudes, indicating higher a-amplitudes in men than women; *p* < 0.04; see Table 2.

**Table 2:** ERG results for summer and winter in subjects with cataracts and in pseudophakes.

	Winter	Summer	Winter	Summer
		
	Cataracts		Pseudophakes	
Scotopic −25 dB b-wave amplitude (μV)^a^	**179.3 (51.9)**	**183.61 (48.7)**	**205.5 (53.0)**	**217.2 (49.6)**
Implicit time (ms)^a^	**104.1 (10.3)**	**107.3 (6.4)**	**97.6 (9.8)**	**97.6 (8.3)**
Scotopic 0 dB a-wave amplitude (μV)^a^	**118.1 (36.6)**	**120.2 (26.5)**	**132.1 (42.0)**	**139.8 (44.6)**
Implicit time (ms)^a^	**20.9 (2.8)**	**21.2 (2.6)**	**18.2 (1.7)**	**18.9 (2.0)**
Scotopic 0 dB b-wave amplitude (μV)	298.0 (88.1)	316.5 (77.3)	308.6 (77.9)	324.4 (95.4)
Implicit time (ms)^a^	**52.4 (2.9)**	**52.5 (2.5)**	**50.6 (2.3)**	**50.6 (2.6)**
Photopic 0 dB a-wave amplitude (μV)	24.6 (6.0)	22.6 (7.2)	24.9 (6.9)	25.6 (8.3)
Implicit time (ms)	15.8 (0.8)	16.0 (0.7)	15.6 (0.8)	15.6 (0.7)
Photopic 0 dB b-wave amplitude (μV)	83.8 (22.6)	83.5 (21.9)	89.1 (23.6)	84.6 (25.7)
Implicit time (ms)	32.1 (1.2)	32.2 (1.2)	32.0 (1.7)	31.4 (1.1)
Flicker 30 Hz amplitude (μV)	73.9 (19.6)	71.5 (21.7)	76.4 (21.5)	73.8 (23.6)
Implicit time (ms)	27.3 (3.0)	27.7 (1.6)	27.4 (1.6)	26.8 (1.2)

*Mean right and left eyes (± SD in brackets); amplitudes are indicated in microvolts and implicit times in milliseconds; *n* = 48*.

*^a^*p* < 0.05; significant differences between cataracts and pseudophakes (in bold)*.

### Pupil Results

**Figure 1:**
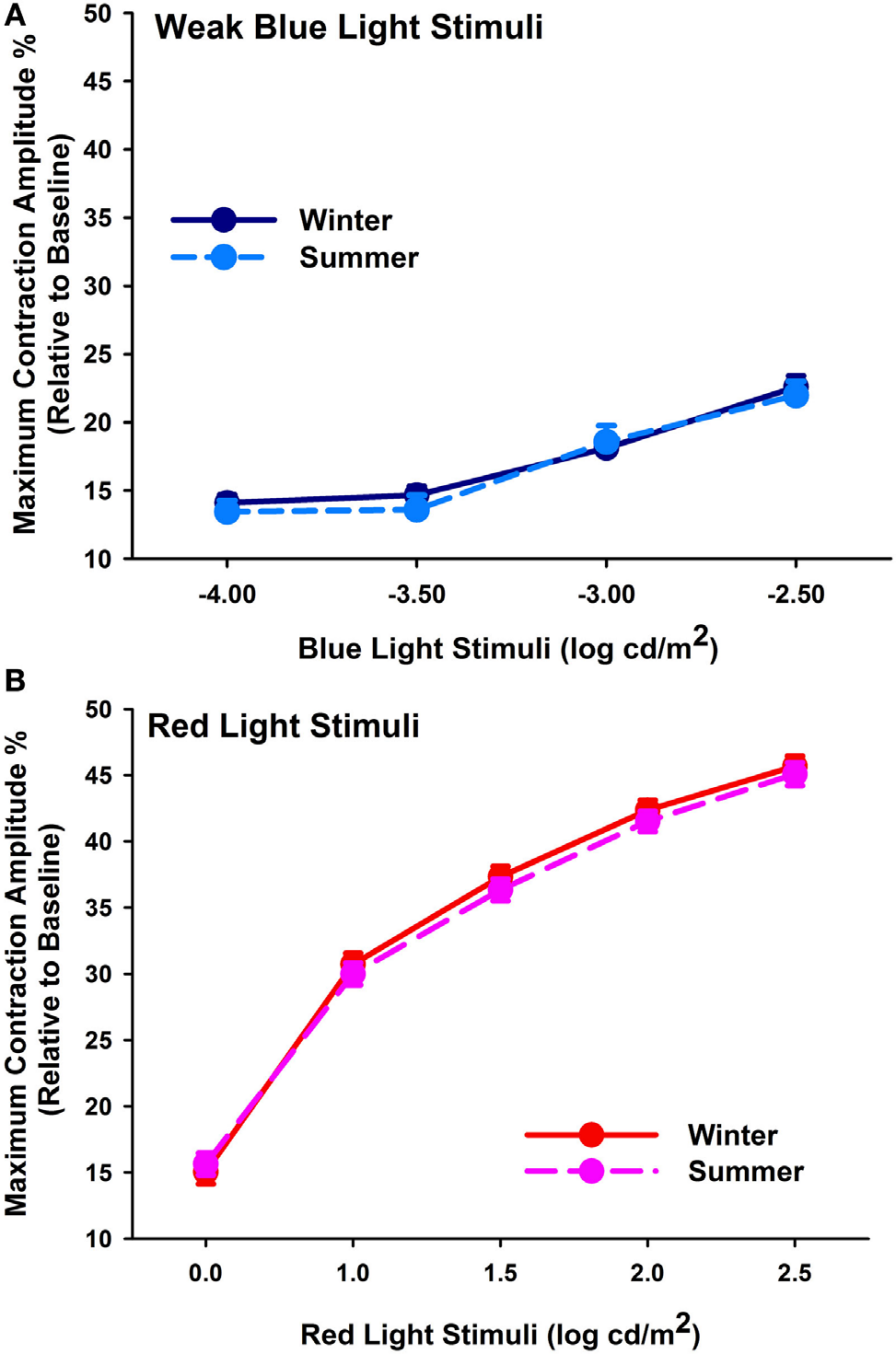
**(A)** Maximum contraction amplitude (%) for the four first rod-weighted weak blue-light stimuli (mean, SEM; *n* = 44) in winter (dark blue symbols, solid line) and summer (light blue symbols, dashed line). **(B)** Maximum contraction amplitude (%) for cone-weighted bright red-light stimuli (mean, SEM) in winter (red symbols, solid line) and summer (pink symbols; dashed line; *n* = 46).

Baseline pupil sizes were larger when measured under scotopic than photopic conditions, and overall, they were larger in winter than summer (*p* < 0.001; Table 3, A; *n* = 52). Cataracts had larger baseline pupil sizes than pseudophakes (*p* = 0.047), and the difference was, on average, 0.36 mm for both photopic and scotopic conditions in the summer and winter recordings. From the rod-weighted light sequence, increasing brightness of the blue-light stimuli led to increasingly greater maximal contraction amplitude (main effect of light stimulus; *F*_3,300_ = 60.5; *p* < 0.0001), without significant differences between the two groups (*p* = 0.25), season (*p* = 0.22), or an interaction between these factors (*p* > 0.37). The maximal contraction amplitude to the dimmest four blue-light stimuli demonstrated no seasonal variation either (*F*_1,43_ = 0.46; *p* = 0.50; *n* = 44; Figure 1A). The covariate age became significant, showing a greater contraction amplitude in the younger participants, i.e., from median splitting those younger than 68.5 years compared with the older ones, i.e., those older than 68.5 years in both groups (*p* = 0.0004).

**Table 3:** (A) Baseline pupil size under light (photopic) and dark (scotopic) adaptation and (B) post-illumination pupil response (PIPR) determined from the melanopsin-weighted sequence.

(A)				
	Photopic^a^		Scotopic	
Mean (SD)	Cataracts^b^	Pseudophakes	Cataracts	Pseudophakes
Winter^c^	4.3 (0.1)	4.0 (0.2)	5.3 (0.2)	4.9 (0.2)
Summer	3.6 (0.1)	3.3 (0.1)	4.4 (0.2)	4.0 (0.2)

**(B)**				
	**Cataracts**		**Pseudophakes**	
	**Mean PIPR % (SD)**	**Mean PIPR % (SD)**	**Mean PIPR % (SD)**	**Mean PIPR % (SD)**

**Monocular blue-light luminance**	**Winter**	**Summer**	**Winter**	**Summer**
1.0 log cd/m^2^	10.9 (11.0)	11.0 (7.1)	15.9 (9.6)	13.4 (8.2)
1.5 log cd/m^2^	23.9 (9.6)	21.3 (10.7)	27.7 (11.0)	26.7 (11.1)
2.0 log cd/m^2^	31.6 (10.7)	33.3 (8.3)	34.0 (10.7)	34.0 (9.2)
**Binocular red-light luminance**	**Winter**	**Summer**	**Winter**	**Summer**
2.3 log cd/m^2^	11.5 (4.8)	13.0 (6.4)	10.0 (9.9)	9.6 (6.6)
**Binocular blue-light luminance**	**Winter**	**Summer**	**Winter**	**Summer**
2.3 log cd/m^2^	46.9 (9.0)	45.5 (6.4)	47.6 (8.9)^d^	41.3 (9.9)

*(A) Scotopic and photopic baseline pupil sizes were calculated as average absolute pupil sizes of the first 0.25 s before the first light stimulus for all participants [in mm (SD); *n* = 52]. Scotopic baseline pupil sizes were larger than photopic pupil sizes = ^a^*p* < 0.0001 and both were larger in winter than summer = ^c^*p* < 0.0001; as well as larger in cataract patients than pseudophakes = ^b^*p* < 0.05. (B) Post-illumination pupil responses [PIPR; %; mean (SD)] for monocular blue light at three different light intensities, and binocular red and blue-light stimuli for winter and summer and cataracts (*n* = 28) and pseudophakes (*n* = 19). Seasonal differences were found only in the binocular PIPR to blue light in pseudophakes = ^d^*p* < 0.05*.

In response to the bright red-light stimuli of the cone-weighted light sequence, maximal contraction amplitudes became larger with increasing stimulus intensity (main effect of light stimulus; *F*_4,410_ = 916.57; *p* < 0.0001; *n* = 46, Figure 1B). There was no significant seasonal (*p* = 0.13) or group difference (*p* = 0.06), or an interaction with the factors group, season, or light stimulus (*p* > 0.61). The covariate age again impacted on the results, with greater responses in younger than older participants (*p* = 0.0003).

**Figure 2:**
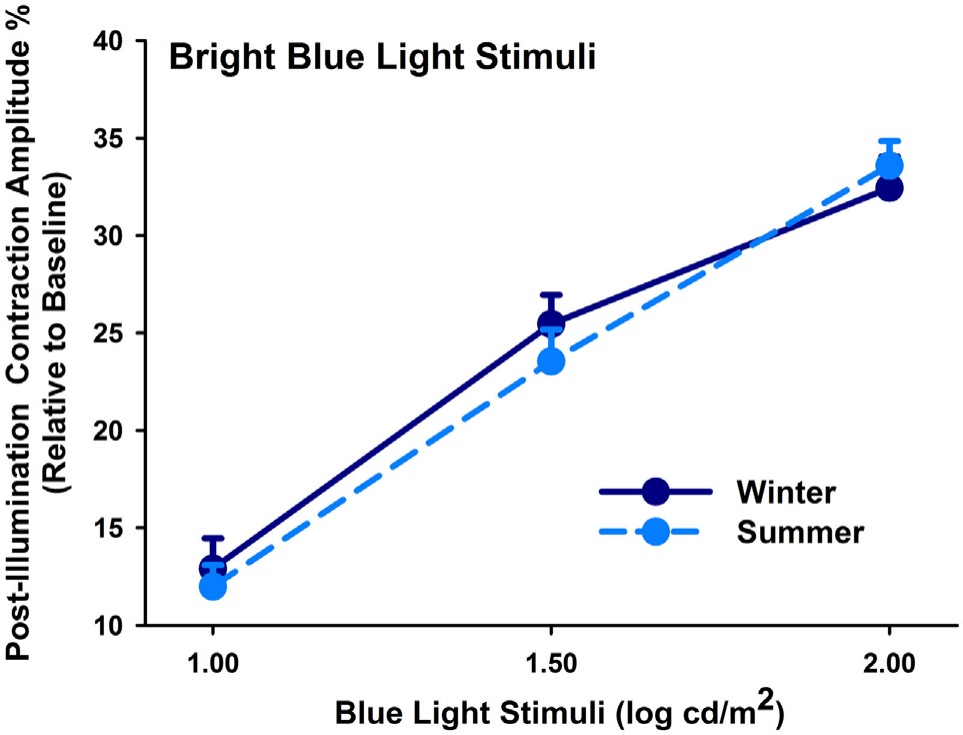
Contraction amplitudes of post-illumination pupil responses (= PIPR; %) for winter (dark blue symbols, solid lines) and summer (light blue symbols; dashed line) seasons (mean, SEM; *n* = 47; monocularly presented).

From the melanopsin-weighted sequence with monocular stimulation, we found increasing PIPR with increasing stimulus intensity (main effect of light stimulus; *F*_2,231_ = 192.58; *p* < 0.0001). The PIPR derived from monocular stimulation exhibited no seasonal (*p* = 0.44) or group differences (*p* = 0.17; Figure 2) or an interaction between these factors (*p* > 0.35).

**Figure 3:**
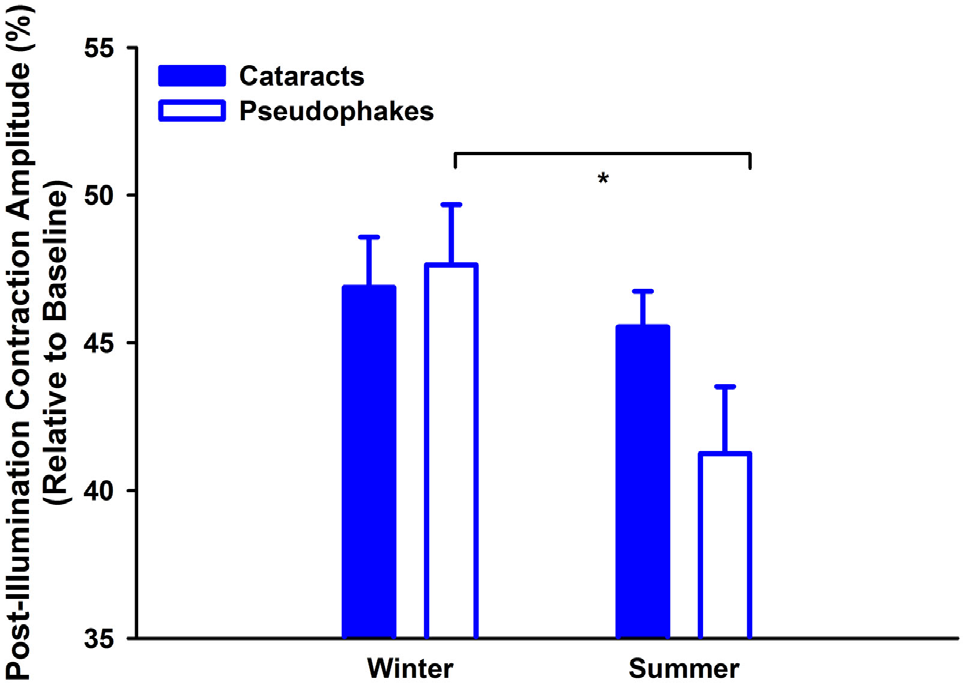
Contraction amplitudes of post-illumination pupil responses (= PIPR; %) for melanopsin-weighted bright blue-light stimuli (2.3 log cd/m^2^; binocularly presented; mean, SEM; *n* = 47). They are shown for both seasons (winter: left side; summer: right side) and both groups (cataracts vs. pseudophakic patients). *p < 0.05 between pseudophakes winter and summer.

Lastly, determination of seasonal differences was carried out with respect to the melanopsin-weighted sequence with binocular stimulation: the pupillary post-illumination dynamics in response to the red-light stimulus was used as a control, as red light at this intensity is considered to be none-to-minimally significant for stimulating melanopsin (36). The pupil re-dilation following red light was rapid such that the post-stimulus contraction was, on average, 10% without significant differences between groups (*p* = 0.06; Table 3, B), and was overall significantly smaller than that to the predominantly melanopsin-activating blue-light stimulus (main effect of color of light stimulus; *p* < 0.0001). Hereafter the PIPR refers only to the post-light contraction to blue light. There was a significantly greater PIPR in winter than in summer (*p* = 0.002; Figure 3; *n* = 47, Table 3, B). For the PIPR there was also an interaction with the factors “group” and “season” (*F*_1,47_ = 4.67; *p* = 0.036). The seasonal variation in PIPR (greater post-illumination contraction amplitude in winter) was observed in pseudophakes (*p* = 0.001), but not in cataracts (*p* = 0.38). Taken together, seasonal variation with larger PIPR in winter than in summer was exhibited for the melanopsin-weighted blue-light stimulus presented binocularly in pseudophakes but not in cataracts.

### Salivary Melatonin Concentrations

**Figure 4:**
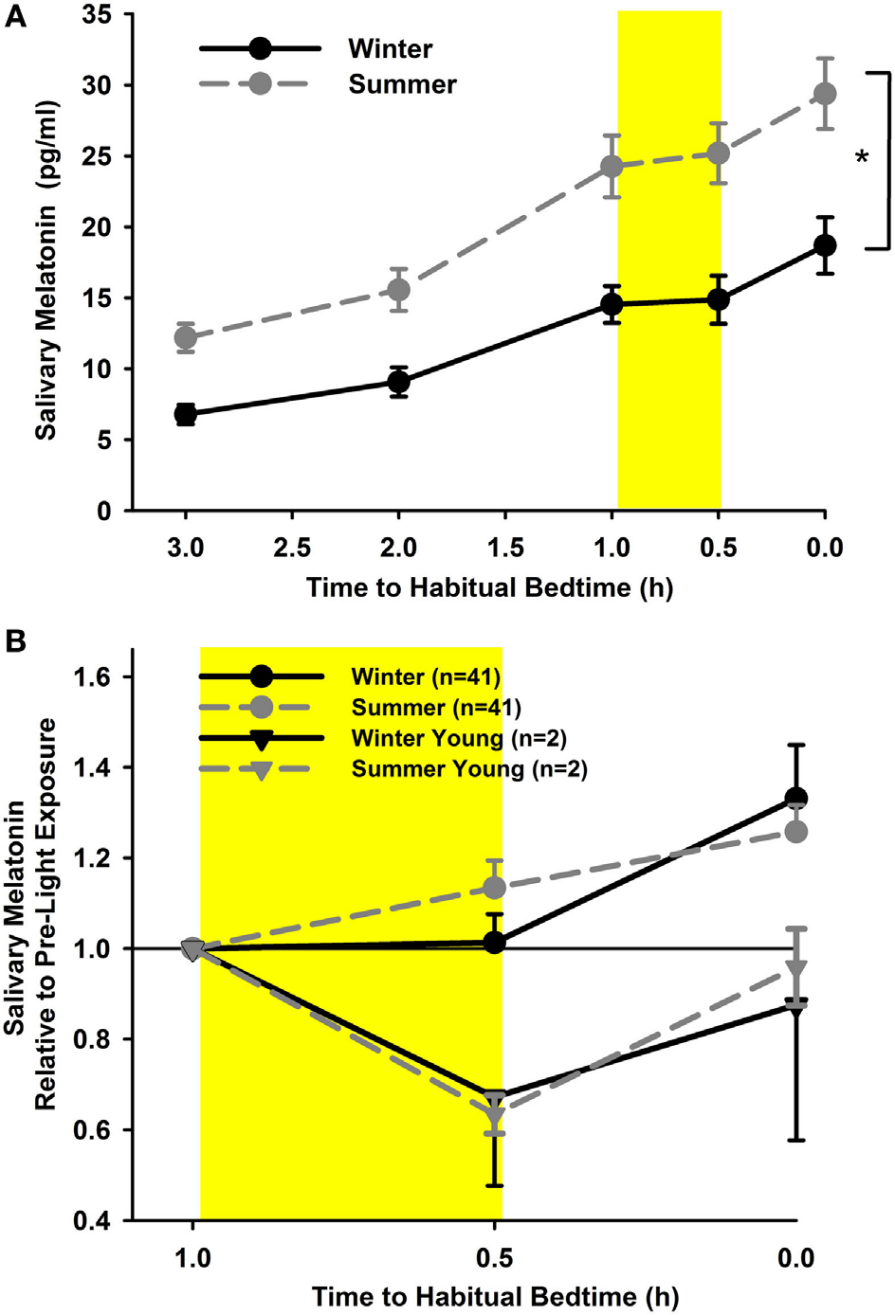
**(A)** Salivary melatonin concentrations for summer (dashed line; gray circles) and winter (solid line; black circles; cataracts and pseudophakes; mean, SEM; *n* = 41). *p < 0.05. **(B)** Relative salivary concentrations (relative to pre-light concentrations; shown as hours relative to habitual bedtime) for both seasons. The yellow rectangle reflects 30 min of light exposure, mean (± SEM). The gray (dashed line) and black triangles (solid line) depict the young controls for summer and winter (*n* = 2).

In order to analyze the melatonin suppression in response to light exposure in the evening, only those participants in whom melatonin concentrations had reached a threshold of 3 pg/ml before light exposure and in whom data from two seasons were available were included in the analysis (*n* = 41). Salivary melatonin concentration was significantly higher in summer than winter (main effect of “season”; with absolute values and including all time points; *F*_1,359_ = 158.0; *p* < 0.0001; Figure 4A). The covariate “iris color” was also significant, indicating that participants with brown eyes (*n* = 18) had lower melatonin concentrations than those with light eyes (*n* = 23; *p* = 0.04).

When expressing the concentrations during and after light exposure (two-time points) relative to the pre-light exposure concentration, there was no significant difference between seasons or groups (main effect of season *F*_1,201_ = 2.45; *p* = 0.12; main effect of group *F*_1,41_ = 3.01; *p* = 0.09; Figure 4B), nor were there any interactions between these factors (*p* = 0.34). During the 30 min of light exposure, melatonin concentrations did not rise but were not significantly suppressed either (*p* = 0.98). However, after light exposure, the concentrations immediately rose again, when compared with pre-light exposure and when compared with melatonin concentrations during light exposure (main effect of time point; *F*_2,202_ = 8.49; *p* = 0.0003). For proof of light-induced suppression of nocturnal melatonin using this protocol, we had collected melatonin samples from two young participants (both age 25 years) who assisted with data collection during the study. There was clear suppression of melatonin during acute light exposure with a mean suppression of 33% during winter and 37% during summer (*n* = 2).

Taken together, there was higher melatonin secretion in summer than in winter, but differences in acute changes of melatonin secretion in response to light exposure at night were not detectable between seasons or groups.

### Subjective Sleepiness, Relaxation, Physical Well-being, and Mood

**Figure 5:**
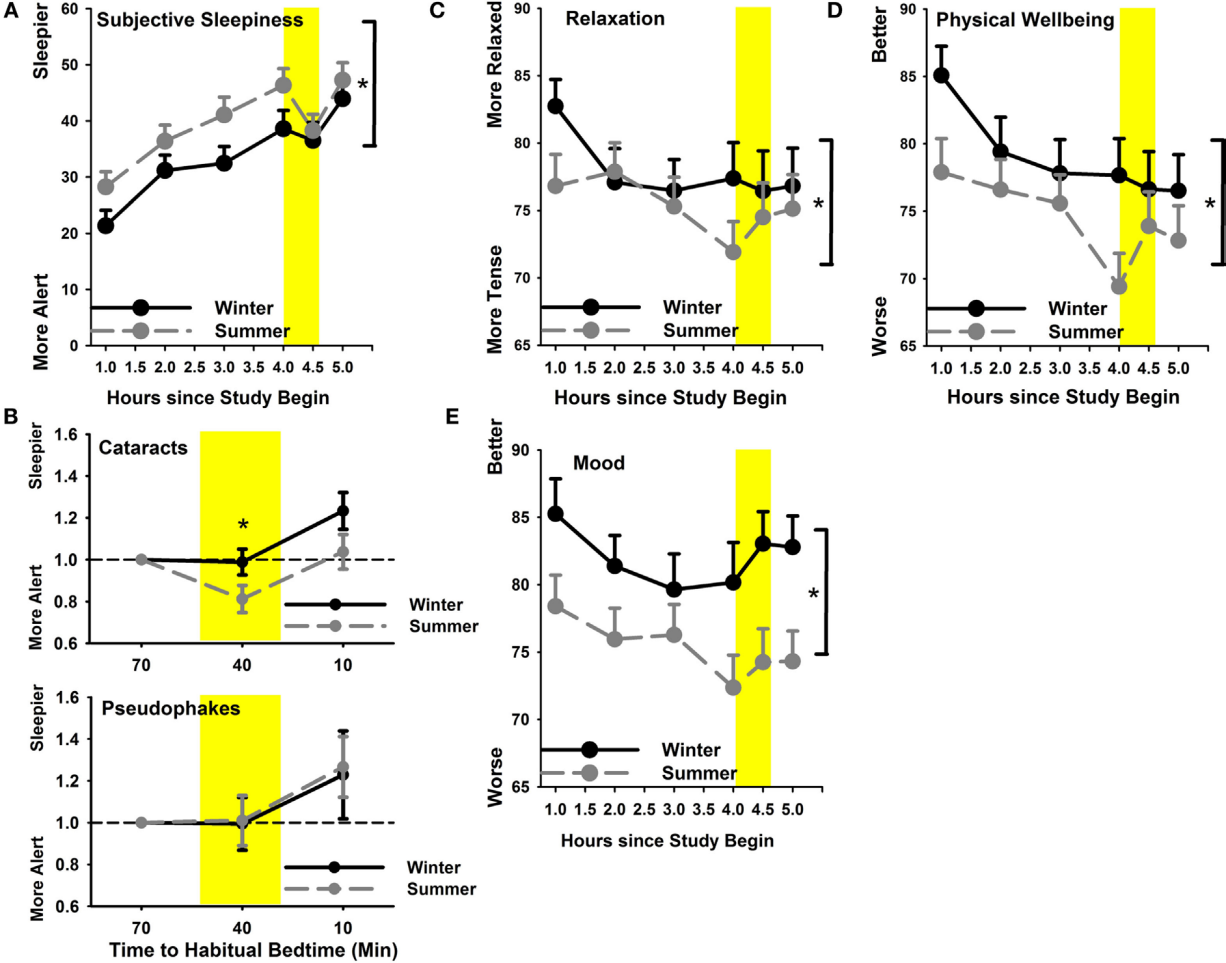
**(A)** Subjective sleepiness in summer (gray symbols; dashed line) and winter (black symbols; solid line); **(B)** subjective sleepiness relative to pre-light exposure for cataracts (upper graph) and pseudophakes (bottom) for both seasons separately; **(C)** relaxation and **(D)** physical well-being; **(E)** mood. *p < 0.05; mean ± SEM, *n* = 47.

Subjective sleepiness was significantly higher in summer than in winter (main effect of season *F*_1,517_ = 21.0; *p* < 0.0001) without a statistical significant group effect (*p* = 0.87). As expected, sleepiness in the evening increased as a function of the number of hours since wake time until the light exposure started (main effect of time *F*_5,517_ = 25.96; *p* < 0.0001; Figure 5A). Relative to pre-light exposure, only cataracts became less sleepy (more alert) in response to light exposure in the summer (group × season; *F*_1,141_ = 4.5; *p* = 0.036; Figure 5B). In addition to increasing sleepiness throughout the course of the evening, all participants reported feeling less relaxed and less physically well until the beginning of light exposure (main effect of time *F*_5,517_ > 2.6; *p* < 0.024). This subjective feeling of poorer relaxation and poorer well-being was significantly greater in summer than winter (main effect of season; *F*_1,517_ > 14; *p* < 0.0003; Figures 5C,D), without a significant group difference (*p* > 0.06). Relative to the last pre-light exposure assessment in the evening, significantly greater relaxation and physical well-being during, and shortly after light exposure was reported in summer (main effect of season; *F*_1,141_ > 6.3; *p* < 0.014) without differences between the two groups (*p* > 0.58).

Mood showed a trend toward slight variation during the evening (main effect of time; *p* = 0.06) and was significantly better in winter than in summer (main effect of season; *F*_1,517_ = 37.68; *p* < 0.0001; Figure 5E) without differences between cataracts and pseudophakes (*p* = 0.27; or an interaction between these factors; *p* = 0.45).

### Rest-Activity Cycles and Sleep

From the analysis of the parameters of rest-activity cycle (see Materials and Methods), we found a significant seasonal variation for IV. Both groups showed higher IV in winter than in summer (main effect of season; *F*_1,86_ = 10.37; *p* = 0.002; *n* = 43; Table 4). For the remaining rest-activity parameters (IS, L5on, M10on, relative amplitude), there were no significant group or seasonal differences (*p* > 0.16).

**Table 4:** Mean values for variables from the rest-activity recordings.

Season	Group	Variables					*N*
		Rel. Amp.	L5on	M10on	IS	IV^a^	
Winter	Cataracts	0.910 (0.009)	24:43 (0:13)	8:40 (0:17)	0.60 (0.018)	0.812 (0.039)	24
Summer		0.899 (0.011)	24:38 (0:13)	8:30 (0:14)	0.63 (0.021)	0.677 (0.028)	24
Winter	Pseudophakes	0.911 (0.012)	24:28 (0:19)	8:25 (0:22)	0.63 (0.022)	0.844 (0.040)	19
Summer		0.901 (0.011)	24:41 (0:19)	8:35 (0:20)	0.62 (0.020)	0.753 (0.026)	19

*Mean (± SEM in brackets; *n* = 43)*.

*Rel. Amp., relative amplitude; L5on, beginning of the 5 h with the least activity (normally during sleep; clock time; hh:mm); M10on, beginning of the 10 h with the greatest activity (normally during waking hours; clock time; hh:mm); IS, inter-daily stability; IV, intra-daily variability*.

*^a^Seasonal difference (main effect of season; *p* = 0.002)*.

Analysis of the sleep periods during the pre-study week revealed similar bed- and wake times, as well as similar times in bed- between groups and between seasons (see Table 5). Despite the similar timing of sleep and wake periods in the two groups, activity watch-derived sleep variables indicated significantly poorer sleep in cataracts than pseudophakes, with cataracts having less sleep (approximately 22 min less on average; main effect of group *F*_1,86_ = 5.16, *p* = 0.03), and greater activity during the night (*F*_1,86_ = 8.4; *p* = 0.005). This was also reflected in a lower sleep efficiency, i.e., the time in bed when participants were sleeping in cataracts compared with pseudophakes (*F*_1,86_ = 6.37, *p* = 0.01).

**Table 5:** Sleep variables from rest-activity recordings.

	Cataracts	Pseudophakes
Bedtime (clock hour; hh:mm)	23:08 (00:06)	23:07 (00:08)
Wake time (clock hour; hh:mm)	7:13 (00:07)	7:23 (00:09)
Time in bed (hh:mm)	8:05 (00:07)	8:16 (00:08)
Actual sleep time (hh:mm)^a^	**6:29 (00:07)**	**6:51 (00:08)**
Actual sleep (%)^a^	**81.67 (1.01)**	**83.90 (0.68)**
Actual wake time (hh:mm)	1:28 (00:05)	1:19 (00:04)
Actual wake (%)	18.34 (1.00)	16.10 (0.68)
Sleep efficiency (%)^a^	**80.27 (1.02)**	**82.79 (0.69)**
Sleep latency (hh:mm)	0:07 (00:01)	0:06 (00:01)
Mean activity score^a^	**18.08 (1.28)**	**14.46 (0.89)**
Mean activity score in active periods^a^^,^^b^	**123.64 (5.68)**	**116.60 (7.41)**
Fragmentation index	33.45 (1.89)	25.15 (1.31)

*Sleep variables from analysis of rest-activity cycles averaged for both seasons and shown for cataracts and pseudophakes (*n* = 43; mean ± SEM). Significant differences are shown in bold*.

*^a^p < 005 between cataracts and pseudophakes*.

*^b^Difference between summer and winter (12.2% higher in winter than summer)*.

The only sleep parameter which showed a seasonal variation was the activity score during nocturnal wake episodes (i.e., activity within the sleep period), which was 12.1 ± 6.3% greater in winter than summer (main effect of season; *F*_1,86_ = 5.06, *p* = 0.03). Other than that, there was no significant seasonal variation (or an interaction with the factor group; *p* > 0.05). Data about habitual light exposure from the light sensor of the activity watches deemed unreliable—since many subjects had inadvertently covered the sensor, especially during the winter season. Analysis of the available light data (29% of all 24-h recordings in winter, and 65% in summer) revealed no significant differences between both groups (*p* > 0.7), but significantly higher light exposure in summer than winter [*p* < 0.05; mean illuminance between 8:00 and 18:00 in summer: 1169 lx (both groups) and winter: 128 lx].

## Discussion

Retinal light exposure mediates the pupil responses via retinotectal projections and melatonin secretion via the retino-hypothalamic tract and polysynaptic signaling to the pineal gland. The common neuronal substrate of both these functions is melanopsin, the photopigment of ipRGCs which modulates these and other light-dependent acute and circadian light effects. The existing literature on diurnal and seasonal variation of melanopsin-dependent functions in non-depressed humans, though limited, has shown both 24 h and winter–summer variation in the pupil responses to bright blue light (47–49) and in secretion profiles of melatonin (67). In this study, we aimed to determine the effects of the differing seasonal light and photoperiod on both melanopsin-dependent variables (pupil and melatonin) in the same healthy participants. Additionally, we examined the effect of cataracts which reduce light transmittance to the retina, particularly the shorter wavelengths of the visual light spectrum, with potential repercussions on the pupil light reflex, melatonin suppression, sleep, and behavior.

We first discuss the findings from the melanopsin-dependent variables and then examine other clinical and behavioral parameters.

### Seasonal Differences of the PIPR

In this study, we found that healthy, non-depressed older adult participants with pseudophakic (non-blue-blocking) lenses demonstrated melanopsin-mediated pupil responses (PIPR) that were greater in winter than in summer, indicating greater responsiveness of intrinsic phototransduction to light in the darker season. The cataract patients did not show this seasonal variation of PIPR. This may be related to the lenticular absorption of blue light, leading to a change in the composition and intensity of light, particularly in summer, such that at the level of the retina, the transmitted ambient light is at lower intensity, and blue-light portion. Daylight exposure in winter on the northern hemisphere has lower blue-light content than in summer (1) and there was no significant difference in the winter PIPR of our subjects with cataracts vs. pseudophakic subjects. Ostrin et al. have shown that the PIPR increases after 2 weeks of reduced blue light (by providing orange tinted goggles to their subjects), presumably an adaptation in the gain of the melanopsin system (68).

The greater PIPR in winter in this study was observed when using a bright blue-light stimulus presented binocularly but not monocularly. We have previously noted a differential binocular vs. monocular stimulus effect of the post-illumination pupil dynamics in patients with bilateral ischemic optic neuropathy (69). In these patients with retinal ganglion cell loss in each eye, the monocular PIPR was reduced as expected but the binocular PIPR was preserved. We speculate a central threshold effect related to binocular summation (70). However, the reasons for the difference in seasonal variation of PIPR from monocular vs. binocular light stimulation are not elucidated and further investigations are needed.

Unexpectedly, the direction of the seasonal variation (greater PIPR in winter) is in contrast to results that we had recently reported in a group of 37 healthy participants who demonstrated greater PIPR in summer (49), and to results from a recent study with a mixed group of healthy adults and patients suffering from major depression (42). We believe these conflicting results in seasonal variation of PIPR may be the influence of several factors including age, prior light exposure, and methodology. Compared with our previous study, the age of participants in the current study cohort was significantly older. In addition, they were semi-retired or retired. We can hypothesize that many of them were spending significantly more time outdoors in summer compared with the younger cohort of the previous study who were hospital workers and thus mostly not exposed to summer light during the day. A third factor may be the light stimulus. In our previous study, a portable pupillometric device with a narrower field of retinal light stimulation was used for monocular stimulation. The results of the current study are based on binocular stimulation with brighter blue-light stimuli and a greater field of stimulation. We thus re-analyzed the PIPR in a subgroup of our subjects selected for age less than 60 years (*n* = 6). We calculated their PIPR evoked from the brightest monocular blue-light stimulus (2.0 log cd/m^2^) and indeed found a slightly greater PIPR in summer (2.1%), even though the difference was not statistically significant (*p* = 0.1). While we have found seasonal differences in PIPR, we acknowledge that this finding is not universal. One previous study reported no seasonal variation in healthy adults (71). Two other studies found seasonal differences: one study of a mixed group of healthy and depressed adults showed greater PIPR in summer (42), and another study of SAD patients had lower PIPR only in winter when compared with controls (46). The distinction whether a larger PIPR indicates greater melanopsin-dependent light sensitivity is important and a common methodological consensus will be needed if the pupil is targeted to be a biomarker of pathology such as seasonal depression or multiple sclerosis (42, 72). Taken together it might be that various other processes related to opsin regulation have variable influence on changes in seasonal light responses. As such, seasonal variation of PIPR may be more a function of any one of these influences rather than a true reflection of the sensitivity of melanopsin photoreception and further investigations are needed to clarify this point.

One such influence on PIPR may be prior light history. Prior light history can modulate the magnitude of acute light responses, such as melatonin suppression in the evening (73–76) or alertness and cognitive performance (77). Depending on the intensity of the daytime light exposure prior to testing, Hébert et al. found that the amplitude of melatonin suppression in response to bright light exposure in the evening was influenced by the amount of light exposure during the morning. Melatonin suppression was attenuated after bright light in the morning and increased after dim light in the morning (73, 74). In non-experimental conditions, prior light history can be related to daylight and artificial light exposure (51). Behavioral habits, such as spending more time outside, especially during daylight hours which are up to 1,000-fold brighter than indoor lighting conditions, in summer than winter and lead to large differences in average daily light exposure during summer vs. winter. Older participants, for example, have been shown to have a greater average duration of light exposure to daylight (without seasonal differences) than younger participants; this is likely related to working hours (78).

In this study, we found that the photopic baseline pupil size was larger in winter, even in the younger subgroup (<60 years; *n* = 6): again, this finding contradicts our previous study (49) in which photopic baseline pupil size was larger in summer. Taken together, the larger pupil size of both studies was noted in the season of the larger PIPR. At first glance, this might suggest that pupil size can mechanistically influence the magnitude of pupil light response simply because of a greater photon flux reaching the retina in eyes with larger pupils. Indeed, Nissen et al. showed a significant difference in PIPR (defined as post-stimulus pupil area under the curve) obtained from the same blue light presented to a pharmacologically dilated pupil vs. a pharmacologically contracted pupil (79). However, the seasonal difference in the baseline photopic pupil size in our participants was quite small (mean difference 0.7 mm) and it did not approach the large difference in pupil size as obtained from pharmacologically manipulated pupils. Thus, an anisocoria less than 1 mm would not be expected to sufficiently explain the seasonal difference in PIPR. Joyce et al. confirmed that normalizing pupil metrics to baseline size, as we have done in calculating relative pupil size, eliminates an anisocoria effect of PIPR (80). It is more likely that this seasonal variation of the light-adapted baseline pupil size is an independent reflection of the seasonal variation in baseline pupil size *per se* and possibly modified by age.

### Seasonal Differences in Melatonin Production and Suppression

Absolute melatonin secretion in the evening was higher in summer than in winter in both groups, similarly to what was reported from melatonin concentrations in post-mortem pineal glands (81). This was not related to any significant shifts in the pattern of sleep-wake cycles between summer and winter. On average, the participants’ habitual bedtimes were less than 10 min apart between summer and winter. The basis for this seasonal variation in melatonin production is not readily explained. However, the light exposure pattern relative to bed- and wake times was also different in summer and winter and we cannot exclude the possibility that brighter light exposure closer to wake time in summer had a phase advancing effect, with an earlier increase of melatonin production in the evening.

To suppress nocturnal melatonin, we selected 400 lx for 30 min as the light exposure condition. From clinical experience, we thought that 400 lx would be well tolerated by the participants without inducing excessive eye closure or discomfort from ocular surface dryness. In addition, a previous study had demonstrated that 6.5 h of exposure to moderate light intensity starting at 274 lx did robustly suppress melatonin by 50% (82). The duration of 30 min in our study may have, in retrospect, been too short and the intensity with a polychromatic white light source too low, but it was also selected for logistic reasons and by balancing subjective light tolerance with results of previous studies. For example, in post-menopausal women, a monochromatic blue or green light-induced detectable melatonin suppression within 15–30 min after light onset (83). Yet in our study, we did not observe suppression of melatonin concentration but rather inhibition of any additional increase in melatonin concentration during light exposure. Older age would appear to be one determinant for the absence of melatonin suppression in our study (mean: 67 years). We performed the nocturnal melatonin suppression test using these same light conditions on two young (mean age 25 years) control participants. We found that melatonin was indeed suppressed by 33% in winter, and 37% in summer, but of course we did not apply any statistics for comparisons of these two subjects. Regarding the effect of age on nocturnal melatonin suppression by light, published studies have shown divided results. Duffy et al. found a decreased sensitivity to moderate light levels for circadian phase shifts of the melatonin rhythms in older participants compared with younger ones, even though the light stimulus by Duffy et al. was also given for a much longer duration (82). However, the study by Najjar et al. (84) showed no difference in melatonin suppression in older vs. younger participants exposed to monochomatic light exposures of different wavelengths for 60 min (84). It is beyond the scope of this study to comment further on age effects on melatonin suppression as this was not a primary hypothesis. We also note that the difference in melatonin concentration between pre-light and post-light exposure in healthy adults with and without cataracts did not show seasonal variation. A limitation of the study is that we did not perform a control condition with only dim light which would have allowed us to analyze dim light adjusted melatonin suppression.

### Seasonal Differences in Light Responses Mediated by Outer Retinal Photoreceptors

The issue of photoperiodicity of outer retinal photoreceptors (rods and cones) is yet unsettled. Using electro-oculography, one study has suggested that retinal sensitivity may be higher in winter than in summer (85). In another study using ERG, indoor workers were found to have higher dark-adapted retinal sensitivity in winter when compared with outdoor workers (86). Other investigators have not, however, demonstrated seasonal variation in the retinal sensitivity of normal subjects with healthy eyes. Using the b-wave of the dark-adapted ERG, Hébert et al. found rod-mediated luminance response curves did not differ between summer and winter in 21 normal subjects (73, 74). However, in that study, patients with sub-syndromic SAD did show a significantly decreased scotopic retinal sensitivity in winter. Similarly, a study using dark adaptometry found no significant seasonal effect of rod or cone threshold in 12 normal subjects whereas summer–winter differences were detected in SAD patients (17).

While not the primary aim of this study, we also measured rod and cone activity in the outer retina by the ERG and by pupil responses in order to have a comparative basis for our results of melanopsin-mediated light responses. We found no seasonal difference in outer retinal light sensitivity determined the pupil responses to the rod-weighted and cone-weighted light sequences. Similarly, we found no seasonal differences in the scotoptic or photopic electrophysiologic responses (ERG). These two results indicate no effect of long-term light changes, such as those related to the photoperiod, on outer retinal light responsiveness.

### Seasonal Differences in Subjective Assessments and Rest-Activity Cycles and Sleep

Overall, the older participants in our study were less sleepy and more relaxed, while feeling better in the dim light conditions of the laboratory on winter evenings compared with summer evenings. However, it was only in summer that light exposure 1 h before habitual bedtime (400 lx, 30 min) was associated with greater relaxation and improvement in reported physical well-being. For subjective sleepiness, only the participants with cataracts became less sleepy (more alert) after the nocturnal light exposure and this was only evident in summer. These results are difficult to interpret. If there is a sensitization process induced by prior low light history in winter, we would have expected greater responses to light exposure in the evening in winter. One might speculate that the dim indoor light conditions of the laboratory in the evening were not unlike the ambient lighting conditions of winter at these hours; thus the evening testing might have been better tolerated and less perturbed in winter than in summer when days are longer and daylight was still present at the moment of entering the laboratory. Likewise, they were more favorably responsive to nocturnal light exposure in summer. It is, however, unclear why participants with cataracts would have a greater alertness from light exposure in summer. Seasonal changes for rest-activity cycles were only found for IV which was greater in winter than summer in both groups. This points to changed rest-activity patterns in both groups in winter—probably a function of less time spent outside.

It has been previously shown that patients with cataracts report lower sleep quality before lens replacement, when compared with after lens replacement (87–90). In addition, the transition from cataractous lens to pseudophakia results in later timing of sleep and melatonin production (91), and faster responses in cognitive tests (55). We also found that participants with cataracts had poorer subjective sleep quality and objective sleep efficiency (assessed by rest-activity recordings 7 days before the study) compared with age-matched pseudophakes. In terms of seasonal variation, unlike the pseudophakes, the participants with cataracts failed to show a winter–summer variation in the melanopsin-mediated pupil response PIPR but they tended toward greater subjective feelings of seasonality. In addition, the participants with cataracts were more responsive to nocturnal light exposure in summertime, showing increased alertness when compared with pseudophakes.

### Main Finding of the Study

In summary, the main finding of this study is that seasonal variation in the physiologic responsiveness from predominant melanopsin activation was observed in the pupil behavior but not in the melatonin suppression. Using binocular blue-light stimulation in older participants without cataracts (having non-blue-blocking artificial lens), the PIPR is greater in winter compared with summer. This variation may reflect long-term adaption to environmental light exposure though notwithstanding various other external factors, in particular the conditions of the light stimulus, the age of the subject, and prior light exposure history may influence such variation. One such influencing factor appears to be lens status: age-matched older participants with bilateral cataracts of mild-to-moderate degree did not demonstrate seasonal variation in the PIPR suggesting that chronically lower light exposure from reduced lens transmission may alter the sensitivity of the melanospin system to acute light exposure.

## Ethics Statement

The study was conducted according to the tenets of the Declaration of Helsinki and received authorization from the local ethical board committee for human research for the canton of Vaud of Switzerland. All study participants provided oral and written informed consent.

## Author Contributions

MM and AK designed the study; ML, KH, and SR performed the study; MM, ML, SR, and AK analyzed the data; MM and AK were drafting the manuscript; and MM, ML, SR, KH, and AK revised it critically; MM, ML, SR, KH, and AK approved the final version; and MM, ML, SR, KH, and AK agreed to be accountable for all aspects of the work in ensuring that questions related to the accuracy or integrity of any part of the work are appropriately investigated and resolved.

## Conflict of Interest Statement

The authors declare that the research was conducted in the absence of any commercial or financial relationships that could be construed as a potential conflict of interest.
